# Physical Activity Patterns According to Demographic, Social, and Clinical Correlates Among Breast Cancer Survivors

**DOI:** 10.1002/cam4.70884

**Published:** 2025-05-20

**Authors:** Michael A. Kebede, Charles E. Matthews, Matthew R. Dunn, Natasha R. Burse, Annie G. Howard, Kelly R. Evenson, Melissa A. Troester

**Affiliations:** ^1^ Department of Epidemiology, Gillings School of Global Public Health University of North Carolina Chapel Hill Chapel Hill North Carolina USA; ^2^ Metabolic Epidemiology Branch, Division of Cancer Epidemiology and Genetics National Cancer Institute Rockville Maryland USA; ^3^ School of Nursing University of North Carolina at Chapel Hill Chapel Hill North Carolina USA; ^4^ Carolina Population Center, Gillings School of Global Public Health University of North Carolina Chapel Hill Chapel Hill North Carolina USA; ^5^ Department of Biostatistics, Gillings School of Global Public Health University of North Carolina Chapel Hill Chapel Hill North Carolina USA

**Keywords:** breast cancer, breast cancer survivors, correlates, physical activity

## Abstract

**Introduction:**

Moderate to vigorous physical activity (MVPA) after breast cancer diagnosis is associated with improved survivorship. However, differences in MVPA by race among breast cancer survivors are not well described in population‐based studies.

**Methods:**

We analyzed data from Carolina Breast Cancer Study Phase 3 (*n* = 2994, 50% Black) participants to evaluate the trajectory of MVPA from pre‐diagnosis to 18‐month post‐diagnosis. Participants self‐reported MVPA at baseline (pre‐diagnosis) and 6‐ and 18‐month post‐diagnosis and were classified as having any MVPA (> 0 min/week) or no MVPA. Associations between MVPA and demographic, social, and clinical variables were estimated using multivariable logistic regression.

**Results:**

At baseline, 84.0% of participants reported any MVPA pre‐diagnosis, which dropped to 55.4% at 6‐month post‐diagnosis, then rebounded to 85.1% by 18‐month post‐diagnosis. Among those who had no MVPA pre‐diagnosis, 32.5% and 71.0% became active at 6‐ and 18‐month post‐diagnosis, respectively. Higher income [adjusted odds ratio (aOR) = 1.33, 95% confidence interval (CI) (1.02, 1.74) > $30K vs. < 15K], lower body mass index [aOR = 1.30, 95% CI (1.00, 1.73) < 25 vs. > 30], low area deprivation [aOR = 1.35, 95% CI (1.08, 1.67) vs. high], high area assets [aOR = 1.54, 95% CI (1.23, 1.93) vs. low], and stage I breast cancer [aOR = 1.72, 95% CI (1.22, 2.43) vs. 3 or 4] were associated with any MVPA at 18‐month post‐diagnosis.

**Conclusion:**

We identified several demographic and social correlates of any MVPA at 18‐month post‐diagnosis, and together with established clinical correlates (such as late disease), these factors may contribute to breast cancer disparities.

## 1 Introduction

As of January 2022, there are 4 million breast cancer survivors in the United States (US) [1]. Quality of life following breast cancer varies by cancer stage and treatment [2], but engaging in at least 150 min of moderate to vigorous physical activity (MVPA) may have benefits for long‐term survivorship and quality of life [3, 4, 5]. However, studies have shown that engaging in any form of MVPA among breast cancer survivors is crucial and has survival advantages compared to physically inactive survivors [4, 6]. Numerous studies support the integration of MVPA into breast cancer care and indicate that increased MVPA is associated with a 24%–45% decrease in all‐cause and breast cancer‐specific mortality [3, 4, 7, 8, 9, 10]. However, MVPA varies according to a range of factors, from biological to environmental factors [11, 12, 13, 14, 15, 16]. Previous studies on breast cancer survivors found that correlates such as higher income, college‐level education, lower weight, and White race were significantly associated with higher MVPA participation [17, 18, 19].

Still, notable knowledge gaps remain in our understanding of MVPA patterns among breast cancer survivors. Specifically, prior studies focused largely on White and older populations, which limits the generalizability of study findings [7, 14, 20, 21, 22, 23, 24, 25, 26]. In addition, prior studies provided data at a single time point, rather than multiple time points, which could help understand the role of cancer treatments in MVPA participation [27, 28]. Community‐level correlates are important predictors of MVPA and have been seldom evaluated [29, 30]. One prior cross‐sectional study among breast cancer survivors found that higher neighborhood median income was associated with an increase in Metabolic Equivalent of Task (MET) minutes of physical activity (PA) per week (MET‐min/week) [31], but many other community‐level socioeconomic variables are understudied [32, 33, 34].

This longitudinal study of breast cancer survivors focused on MVPA from pre‐diagnosis to 6‐ and 18‐month post‐diagnosis. Using data from Carolina Breast Cancer Study Phase 3 (CBCS3), a diverse population‐based study that oversamples Black women and younger women (< 50 years old), we assessed temporal changes in MVPA among women with breast cancer and evaluated demographic, social, and clinical correlates related to MVPA patterns.

## 2 Methods

### 2.1 Study Design

CBCS3 is a case‐only prospective population‐based survivorship study of Black and non‐Black women with invasive breast cancer in North Carolina (NC). CBCS3 obtained informed consent and enrolled 2998 women (50% Black) with primary breast cancer diagnosed between May 1, 2008, and October 31, 2013, across 44 NC counties. This study was approved by the Office of Human Research Ethics Institutional Review Board at the University of North Carolina at Chapel Hill. Participants were recruited within 2 months of diagnosis using rapid case ascertainment aided by the North Carolina Central Cancer Registry and Lineberger Comprehensive Cancer Center. Breast cancer diagnoses were confirmed using pathology reports and medical records from treating facilities. Eligibility was restricted to English‐speaking women, aged 20–74 years at the time of diagnosis and diagnosed with stage I–IV breast cancer. We excluded individuals missing MVPA or disease stage data (*n* = 4) at baseline and at follow‐up. Additionally, 54 participants with missing surgery data were excluded only when assessing effect estimates for the clinical variables.

Our conceptualization of race and socioeconomic status (SES) was based on “cells to society” model, which posits that health is driven by multilevel (biological, demographic, clinical, social, and structural) correlates and emphasizes race as a social construct [16]. Participants self‐identified their race based on categories provided or open‐ended responses in the survey. Community‐level SES was defined using census tract information on deprivation and assets, as described under the “correlates section.”

Data were collected by trained nurses and interviewers at regular intervals for up to 10 years post‐diagnosis. At approximately 6‐month post‐diagnosis (mean ± standard deviation; 5.7 ± 3 months), baseline interviews occurred in person, and at 18‐month interviews occurred by phone.

### 2.2 Physical Activity Measurement

Participants reported (1) MVPA within the 3 months prior to breast cancer diagnosis (collected at baseline, mean 5.7 months post‐diagnosis), (2) MVPA 6‐month post‐diagnosis (collected at mean 5.7 months post‐diagnosis), and (3) MVPA 18‐month post‐diagnosis. Questions about PA were patterned on those used in the 2001 Behavioral Risk Factor Surveillance System Survey with established reliability [35, 36]. The pre‐diagnosis question was phrased as, “In the three months prior to your diagnosis of breast cancer, aside from any work you did at a job, did you do moderate physical activities?” whereas the 6‐month post‐diagnosis MVPA questionnaire was phrased as, “In the past seven days, aside from any work you did at a job, did you do moderate physical activities?” If participants responded “yes,” they were asked about the frequency (days per week) and duration (minutes per day) of their PA. MVPA included sports (e.g., bicycling, dancing), leisure activities (e.g., brisk walking, playing with children), housework (e.g., vacuuming, gardening, yard work), and occupational PA (e.g., house cleaner, farm work, teaching, nurse). Similar questions were also asked for vigorous PA at both time periods (pre‐ and 6‐month post‐diagnosis). At all three timepoints (pre‐diagnosis, 6‐month post‐diagnosis, and 18‐month post‐diagnosis), frequency data were available; however, duration data were only available for pre‐diagnosis and 6‐month post‐diagnosis timepoints.

Weekly minutes of pre‐ and 6‐month post‐diagnosis MVPA were categorized as sufficient MVPA (≥ 150 min of PA per week), insufficient MVPA (> 0 to < 150 min of PA per week), or no MVPA reported based on the 2018 PA Guidelines for Americans [37, 38, 39]. We employed an adjusted measure of MVPA (MVPA adj), calculated as 2*vigorous PA + moderate PA. This provides greater weight to vigorous PA and is consistent with 2018 PA Guidelines for Americans [19, 38, 40]. Changes in MVPA (differences between pre‐diagnosis and 6‐month post‐diagnosis) levels were assessed as both continuous (6‐month post‐diagnosis MVPA levels minus pre‐diagnosis MVPA) and categorical: increased (> 30 min), decreased (> 30 min), and no change (within 30 min). In our main analysis, we collapsed MVPA into dichotomous MVPA [any MVPA (> 0 min/week) or no MVPA] and change in MVPA [(increased MVPA or no change in MVPA) or (decreased MVPA)] for simplicity and interpretability.

At 6‐month post‐diagnosis, MET‐min/week were assessed as a continuous variable to measure 6‐month post‐diagnosis MVPA and changes in MVPA. To calculate MET‐min/week, MET values of 4.0 and 8.0 were assigned to moderate‐ and vigorous‐intensity activity, respectively, and were multiplied by the number of minutes per week for each activity type, and then summed. The MVPA data at 18‐month post‐diagnosis were collected to assess days per week of MVPA, whereas pre‐ and 6‐month post‐diagnosis MVPA collected data on both days per week and minutes per day of MVPA. MVPA was categorized into 0 days, 1–3 days, and 4–7 days per week as well as dichotomized into any (> 0 days) versus no (0 days) MVPA.

### 2.3 Demographic, Social, and Clinical Correlates

In this study, the term “correlates” is used to describe variables potentially associated with MVPA and does not imply a statistical correlation or a specific quantitative linear relationship between two continuous variables [41].

Demographic measures included race (Black or non‐Black), age at diagnosis (< 50 or ≥ 50), family income (≤ $15K, > $15 to ≤ $30K, and ≥ $30K), and education (≤ high school [HS] graduate and ≥ some college).

Participants' access to health care was defined using latent class analysis, characterized into either “fewer barriers” or “more barriers” based on probabilities of being uninsured, job loss, financial, and transportation issues [42]. Community‐level SES was defined at the census tract level with latent class analysis using data from the American Community Survey [43]. Briefly, the latent variables “deprivation” (encompassing disadvantageous characteristics such as lack of access to a vehicle, public assistance, unemployment, crowded housing, renting, and being considered below the poverty line) and “assets” (encompassing advantageous characteristics such as household value) were utilized as a dichotomy (low vs. high). Participants were assigned the community‐level deprivation and assets categories for the census tract of their residence at cancer diagnosis.

Clinical measures were collected at cancer diagnosis and abstracted from medical records. These included chemotherapy treatment (received before baseline survey or no), endocrine therapy (yes or no), lymphedema (yes or no), cancer stage (I–IV), and body mass index (BMI: < 25, 25–30, > 30 kg/m^2^).

### 2.4 Statistical Analysis

In this study, we described temporal changes in MVPA among study participants in relation to demographic (such as age, income, and education), social (such as community‐level area deprivation and assets), and clinical correlates (such as cancer stage and chemotherapy). The distribution of all demographic, social, and clinical correlates for the overall CBCS3 cohort and within race and age groups within the cohort were assessed in univariate analyses. We used analysis of variance (ANOVA) tests and Pearson chi‐squared tests to assess racial differences in MVPA by time, and generated *p*‐values for continuous and categorical variables, respectively. We also compared the number of days per week of MVPA at 6‐month post‐diagnosis and 18‐month post‐diagnosis to evaluate differences from pre‐diagnosis levels. Change in MVPA (pre‐ to 6‐month post‐diagnosis) using mean MET‐min/week and mean min/week stratified by race and age, and categorical classification of MVPA (sufficient, insufficient, and no MVPA) was evaluated at different time points.

Logistic regression was conducted to compare those with any MVPA (> 0 min/week of MVPA) at 6‐ and 18‐month post‐diagnosis to those performing no MVPA. These associations between any MVPA and demographic, social, and clinical correlates were estimated in a reduced model adjusted only for age at diagnosis, and in a multivariable model adjusted for age at diagnosis, pre‐diagnosis MVPA, receipt of endocrine therapy, receipt of chemotherapy, cancer stage, and lymphedema. We focused on treatment and clinical characteristics in the multivariable models to account for the role of disease severity on participants' MVPA. However, we included demographic and social characteristic multivariable models to assess how they change effect estimates. Potential confounders between correlates and MVPA participation were identified via literature review followed by exploring multicollinearity. Finally, to improve model parsimony we applied the backward selection method excluding confounders that did not affect the estimate.

Sensitivity analyses were conducted to assess whether correlates were more strongly associated with sufficient MVPA as compared to insufficient MVPA. In these analyses, we used the three‐category MVPA variable including sufficient, insufficient MVPA, and no MVPA. We included the same correlates in a reduced and multivariable polytomous logistic regression model and estimated odds ratios for categories of MVPA [sufficient MVPA or insufficient MVPA vs. no MVPA (reference)].

ANOVA was used to assess the relationship between various correlates and MET‐min/week of total MVPA at 6‐month post‐diagnosis and mean changes in MVPA using the same reduced and adjusted regression models described for logistic regression. These analyses were stratified by race due to interest a priori. The final multivariable‐adjusted model was selected based on prior knowledge of MVPA patterns and backward selection. SAS version 9.4 (Cary, NC) and R version 4.3.3 (Vienna, Australia) were used to conduct all analyses.

## 3 Results

The CBCS3 analytic sample was 49.8% Black, had a mean (SD; range) age at diagnosis of 51.7 (11.1; 23–74) years, and 81.8% were diagnosed with stage I or II breast cancer (Table 1). The distribution of clinical and demographic correlates varied by race and age. Black women in both age categories had less frequent college‐level education, experienced healthcare barriers, had a higher stage at diagnosis, and more frequently received chemotherapy.

**TABLE 1:** Demographic, social, and clinical correlates of CBCS3 participants by race and age (*N* = 2994).

	All ages	20–49 years		50–74 years	
	*N* = 2994	*N* = 1489		*n* = 1505	
	Overall	Non‐Black	Black	Non‐Black	Black
	*N* (%)	*N* (%)	*N* (%)	*N* (%)	*N* (%)
Income					
> 30K	1831 (61.2)	610 (81.3)	390 (52.8)	528 (70.2)	303 (40.2)
> 15K to ≤ 30K	544 (18.2)	73 (9.7)	166 (22.5)	107 (14.2)	198 (26.3)
≤ 15K	619 (20.7)	67 (8.9)	183 (24.8)	117 (15.6)	252 (33.5)
Missing					
Education					
≥ Some college	1179 (39.4)	431 (57.5)	266 (36.0)	293 (39.0)	189 (25.1)
≤ HS graduate	1815 (60.6)	319 (42.5)	473 (64.0)	459 (61.0)	564 (74.9)
Missing					
Access to healthcare					
Fewer barriers	2301 (76.9)	609 (81.2)	542 (73.3)	617 (82.1)	533 (70.8)
More barriers	693 (23.1)	141 (18.8)	197 (26.7)	135 (18.0)	220 (29.2)
Missing					
Community level SES					
Deprivation					
Low	1830 (61.1)	613 (81.7)	338 (45.7)	582 (77.4)	297 (39.4)
High	1164 (38.9)	137 (18.3)	401 (54.3)	170 (22.6)	456 (60.6)
Assets					
Low	1704 (56.9)	334 (44.5)	475 (64.3)	338 (45.0)	557 (74.0)
High	1290 (43.1)	416 (55.5)	264 (35.7)	414 (55.1)	196 (26.0)
BMI					
< 25	726 (24.4)	305 (40.8)	125 (17.0)	210 (28.0)	86 (11.5)
25–29	821 (27.5)	207 (27.7)	177 (24.1)	258 (34.5)	179 (23.8)
> 30	1435 (48.1)	236 (31.6)	432 (58.9)	281 (37.5)	486 (64.7)
Missing	12	2	5	3	2
Endocrine therapy					
Yes	2019 (67.4)	538 (71.7)	416 (56.3)	585 (77.8)	480 (63.8)
No	975 (32.6)	212 (28.3)	323 (43.7)	167 (22.2)	273 (36.3)
Missing					
Chemotherapy					
Yes	1872 (62.5)	520 (69.3)	577 (78.1)	340 (45.2)	435 (57.8)
No	1122 (37.5)	230 (30.7)	162 (21.9)	412 (54.8)	318 (42.2)
Lymphedema					
No	2817 (94.1)	717 (95.6)	679 (92.0)	715 (95.1)	706 (93.8)
Yes	176 (5.9)	33 (4.4)	59 (8.0)	37 (4.9)	47 (6.2)
Missing	1	0	1	0	0
Stage					
I	1225 (40.9)	291 (38.8)	192 (26.0)	422 (56.1)	320 (42.5)
II	1225 (40.9)	331 (44.1)	367 (49.7)	236 (31.4)	291 (38.7)
III or IV	544 (18.1)	128 (17.1)	180 (24.4)	94 (12.5)	142 (18.9)

Abbreviations: BMI, body mass index; HS, high school; SES, socioeconomic status.

**FIGURE 1:**
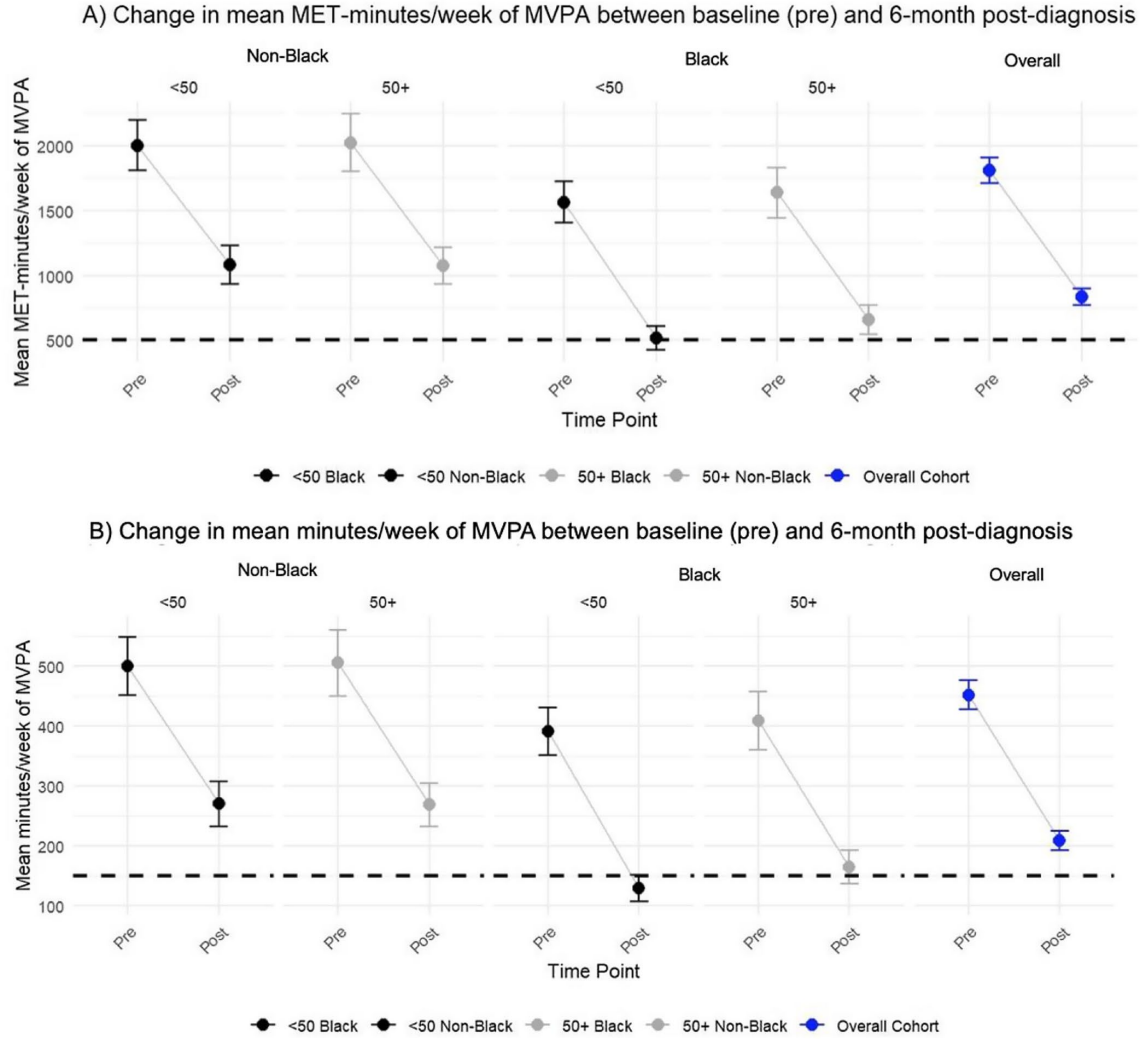
Change in MVPA pre‐diagnosis to 6‐month post‐diagnosis by race and age (A) Change in mean MET‐min/week of MVPA and (B) Change in mean min/week of MVPA. In the dumbbell plot, blue color refers to the overall cohort, black color refers to women < 50 years, and gray color refers to women > 50 years. The horizontal dashed line represents the 2018 Physical Activity Guidelines for Americans of ≥ 150 min/week of moderate physical activity and ≥ 75 min/week of vigorous physical activity. The bars in the dumbbell plot represent the standard errors. Plots are unadjusted estimates of the mean.

Figure 1 shows that on average, participants reported lower levels of total min/week and total MET‐min/week of MVPA at 6‐month post‐diagnosis compared to pre‐diagnosis (Figure 1A,B). Overall, the mean change (95% CI) in MVPA was −974.1 (−1064.4, −883.8) MET‐min/week. At 6‐month post‐diagnosis, the mean MET‐min/week difference (95% CI) between non‐Black and Black women per week was 487.50 (361.1, 613.8). The differences by race were more pronounced in Black women < 50 years than Black women ≥ 50 years. The change in mean MET‐min/week (pre‐diagnosis to 6‐month post‐diagnosis) between non‐Black and Black women across both age groups was not meaningfully different (72.8 [−107.70, 253.3] *p* = 0.42).

**FIGURE 2:**
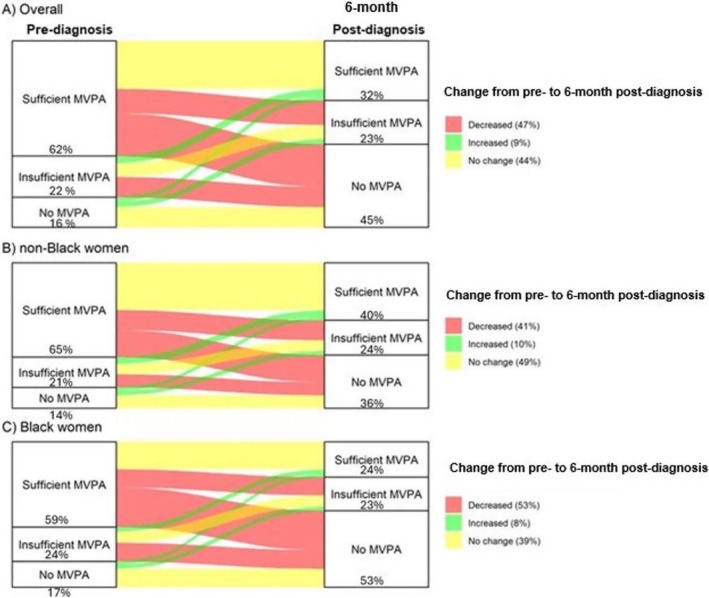
Distribution of MVPA levels of pre‐diagnosis, 6‐month post‐diagnosis, and trajectory of MVPA from pre‐diagnosis to 6‐month post‐diagnosis for (A) all races combined, (B) non‐Black women, and (C) Black women. The flow of the lines indicates whether activity increased (green), decreased (red), or stayed the same (yellow).

Figure 2 shows the temporal change in categorical MVPA using a Sankey diagram. Participants engaging in MVPA declined between pre‐diagnosis and 6‐month post‐diagnosis. This decline was more evident among Black women compared to non‐Black women.

### 3.1 Demographic, Social, and Clinical Correlates and MVPA at 6‐Month Post‐Diagnosis

**FIGURE 3:**
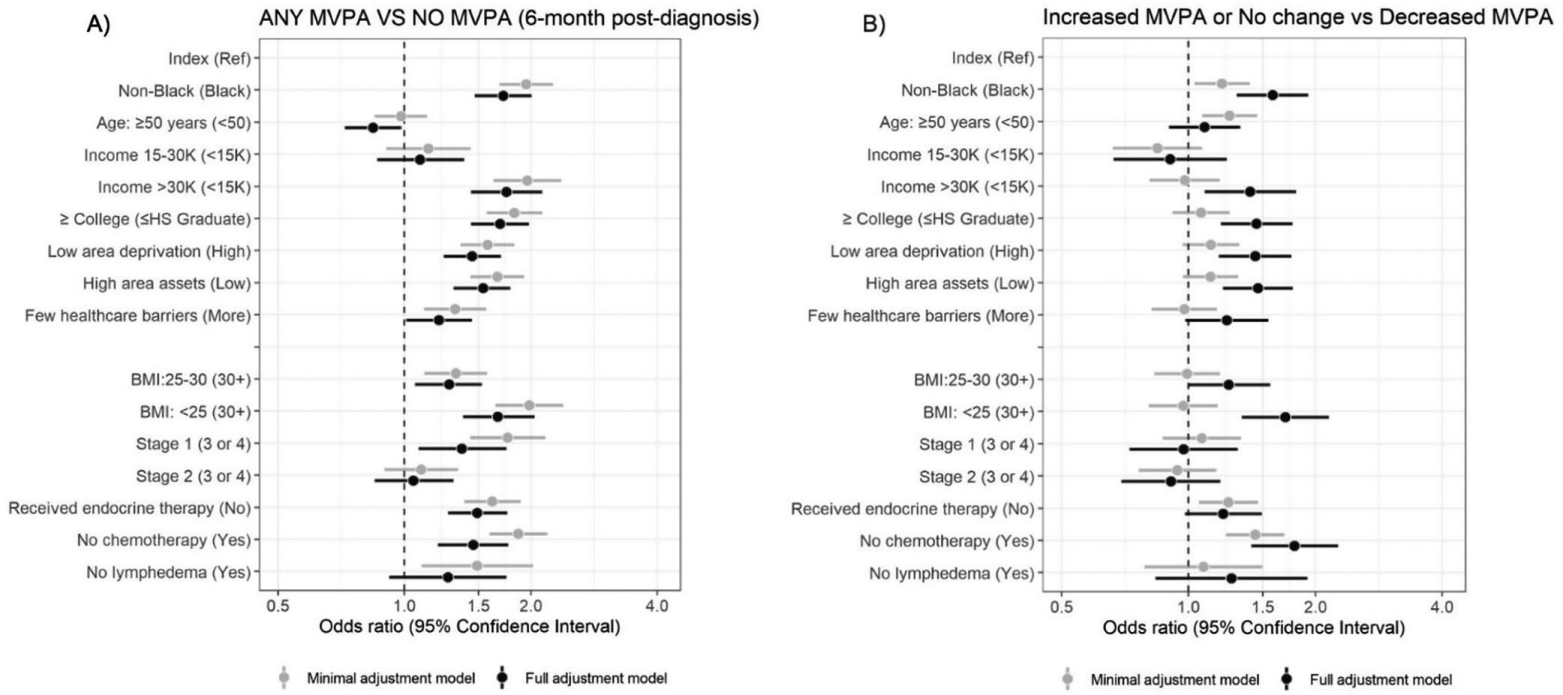
Demographic, social, and clinical correlates in relation to (A) 6‐month post‐diagnosis and (B) change in MVPA since pre‐diagnosis. Model 1 or the minimal adjustment model (gray) is adjusted for age only, and model 2 or the full adjustment model (black) is adjusted for min/week of physical activity pre‐diagnosis, age at diagnosis, endocrine therapy, lymphedema, stage, and chemotherapy where appropriate. Index represents the risk factor that is assessed. BMI, body mass index; HS, high school; MVPA, moderate to vigorous physical activity; Ref, reference.

Considering demographic correlates, in the full adjustment model any MVPA at 6‐month post‐diagnosis was significantly associated with non‐Black race [OR = 1.7, 95% CI (1.4, 2.0)] versus Black race, income > 30K [OR = 1.7, 95% CI (1.4, 2.1)] versus < 15K, and ≥ some college education [OR = 1.7, 95% CI (1.4, 1.9)] versus ≤ high school education (Figure 3A). Similar associations were observed when considering temporal patterns (increased or no change vs. decreased MVPA, Figure 3B) by these correlates.

Considering social correlates, low area deprivation [OR = 1.4, 95% CI (1.2, 1.7)] versus high area deprivation, high area assets [OR = 1.5, 95% CI (1.3, 1.8)] versus low area assets, and fewer health care barriers [OR = 1.2, 95% CI (1.0, 1.4)] versus more health care barriers were associated with any MVPA in the full adjustment model, and similar associations were observed considering temporal patterns.

Considering clinical correlates, in the full adjustment model, stage I breast cancer [OR = 1.4, 95% CI (1.1, 1.7)] versus cancer stages III or IV, receipt of endocrine therapy [OR = 1.5, 95% CI (1.3, 1.8)] versus no receipt, no chemotherapy [OR = 1.5, 95% CI (1.2, 1.8)] versus chemotherapy receipt, and BMI < 25 [OR = 1.7, 95% CI (1.4, 2.0)] versus BMI 30+ were associated with any MVPA.

In sensitivity analyses, separating any MVPA into sufficient and insufficient did not alter the general directions of association (Tables S1–S3). For example, correlates associated with sufficient MVPA compared to no MVPA at 6‐month post‐diagnosis included: non‐Black race [OR = 2.0, 95% CI (1.7, 2.4)] versus Black race, income > 30K [OR = 2.1, 95% CI (1.6, 2.7)] versus < 15K, BMI < 25 [OR = 2.0, 95% CI (1.6, 2.5)] versus BMI 30+, fewer health care barriers [OR = 1.3, 95% CI (1.0, 1.6)] versus more health care barriers, no lymphedema [OR = 1.6, 95% CI (1.0, 2.4)] versus lymphedema, and cancer stage I [OR = 1.5, 95% CI (1.1, 2.1)] versus cancer stages III or four. Lymphedema and late cancer stage were not associated with increased MVPA.

**FIGURE 4:**
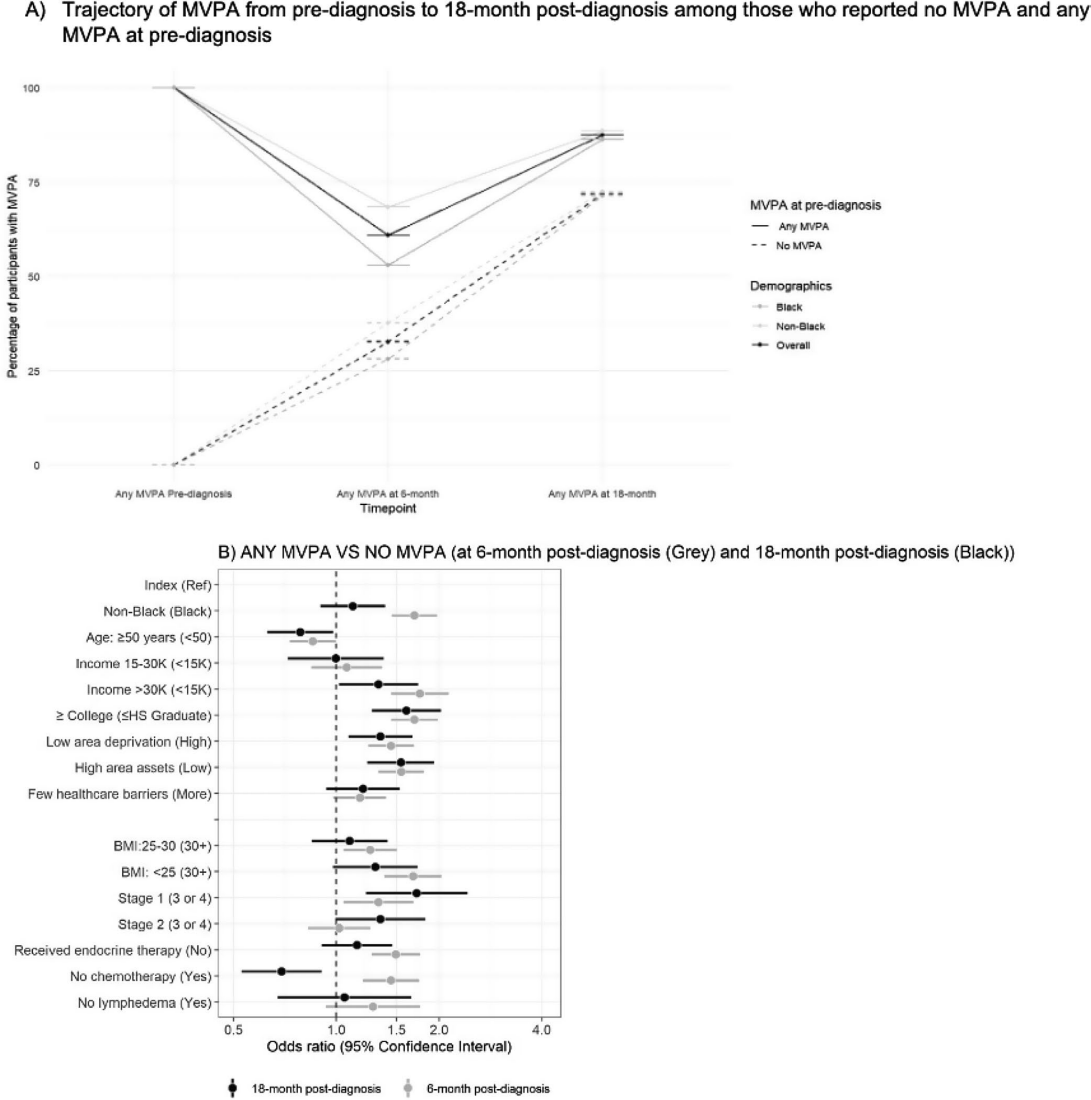
Trajectory and Predictors of MVPA according to (A) Trajectory of MVPA from pre‐diagnosis to 18‐month post‐diagnosis, (B) Association between demographic, social, and clinical correlates related to 6‐ and 18‐month post‐diagnosis (using days per week of MVPA data only). Gray and Black colors represent 6‐ and 18‐month post‐diagnosis, respectively. The solid line represents participants who performed any MVPA pre‐diagnosis, and the dashed line represents participants who performed no MVPA pre‐diagnosis. Both 6‐ and 18‐month post‐diagnosis models are adjusted for min/week of physical activity pre‐diagnosis, age at diagnosis, endocrine therapy, lymphedema, stage, and chemotherapy where appropriate. Index represents the risk factor that is assessed. BMI, body mass index; HS, high school; MVPA, moderate to vigorous physical activity; Ref, reference. The sample size of participants with any MVPA at pre‐diagnosis and 6‐month post‐diagnosis is 2994 and at 18‐month post‐diagnosis is 2759.

Tables S4–S6 report MVPA at 6‐month post‐diagnosis and changes in MVPA associated with the correlates on a continuous scale. We observed similar patterns to our analyses of categorical MVPA: non‐Black race, income > 30K, some college‐level education, low area deprivation, high area assets, and lesser clinical burden were associated with MVPA participation at 6‐month post‐diagnosis. In Tables S7–S9, after modeling for demographic and social characteristics, we found non‐Black race, age ≥ 50, BMI < 25, endocrine therapy receipt, and no chemotherapy receipt were associated with sufficient MVPA at 6‐monthpost‐diagnosis and increased or no change in MVPA. Table S10 complements Figures 1 and 2, which show percent change in MVP, whereas Tables S11–S16 complement Figures 3 and 4B, showing the logistic regression estimates of the forest plots.

### 3.2 MVPA at 18‐Month Post‐Diagnosis

Figure 4 and Table 2 shows proportions of participants with any MVPA over time. Compared to baseline where 84% of participants reported any MVPA, any MVPA dropped to 55.4% at 6‐month post‐diagnosis and then rebounded to 85.1% at 18‐month post‐diagnosis. This U‐shaped trajectory was observed in both Black and non‐Black participants. We observed Black women reported fewer MVPA days per week compared to non‐Black women at each timepoint. Specifically, 82.3%, 47.3%, and 83.8% of Black participants reported participating in MVPA at pre‐diagnosis, 6‐month, and 18‐month post‐diagnosis, respectively, while 86.6%, 63.5%, and 86.3% of non‐Black participants reported MVPA at the same timepoints. Among participants who reported no MVPA at baseline, 30% reported performing any MVPA at 6‐month post‐diagnosis and 70% reported performing any MVPA at 18‐month post‐diagnosis. Significant associations were observed between any MVPA at 18‐month post‐diagnosis and income > 30K [OR = 1.3, 95% CI (1.0, 1.7)] versus < 15K, some college education [OR = 1.6, 95% CI (1.3, 2.0)] versus ≤ high school education, low area deprivation [OR = 1.3, 95% CI (1.1, 1.7)] versus high area deprivation, high area assets [OR = 1.5, 95% CI (1.2, 1.9)] versus low area assets, and stage I breast cancer [OR = 1.7, 95% CI (1.2, 2.4)] versus cancer stages III or IV.

**TABLE 2:** Descriptive statistics of MVPA over time (pre‐diagnosis to 18‐month post‐diagnosis) by race.

	Pre‐diagnosis	6‐month post‐diagnosis	18‐month post‐diagnosis
	*N* (%)	*N* (%)	*N* (%)
Overall (*N* = 2994)			
MVPA days per week			
0 days	480 (16.0)	1334 (44.6)	411 (14.9)
1–3 days	1480 (49.4)	1054 (35.2)	1388 (50.3)
4–7 days	1034 (34.6)	606 (20.2)	960 (34.8)
Missing	0	0	235
Non‐Black (*N* = 1502)			
MVPA days per week			
0 days	216 (14.4)	548 (36.5)	193 (13.7)
1–3 days	746 (49.7)	590 (39.3)	695 (49.3)
4–7 days	540 (35.9)	364 (24.2)	522 (37.0)
Missing	0	0	92
Black (*N* = 1492)			
MVPA days per week			
0 days	264 (17.7)	786 (52.7)	218 (16.2)
1–3 days	734 (49.2)	464 (31.1)	693 (51.4)
4–7 days	494 (33.1)	242 (16.2)	438 (32.4)
Missing	1	1	143

Abbreviation: MVPA, moderate to vigorous physical activity.

## 4 Discussion

In this population of racially diverse women with breast cancer, we found that participants experienced a decline in mean MET‐min/week and mean min/week of MVPA from pre‐diagnosis to 6‐month post‐diagnosis, with more pronounced decreases among Black women. This decrease was transient for most participants, and 85% engaged in some form of MVPA by 18‐month post‐diagnosis. Higher income, college education, low area deprivation, and high area assets were associated with any MVPA at 6‐ and 18‐month post‐diagnosis, but older age was associated with no MVPA at both time points. Treatment correlates (endocrine therapy, stage I breast cancer, and no chemotherapy receipt) were associated with 6‐month post‐diagnosis MVPA, but differences by clinical factors were attenuated at 18‐month post‐diagnosis. The study provides important information on subgroups of cancer survivors that may benefit the most from interventions to improve MVPA.

Our findings were consistent with previous studies in mostly non‐Black participants, showing that participants with a college education, higher income, lower BMI, and fewer healthcare barriers more frequently reported any MVPA [25, 44, 45]. Harrison et al. [26] reported higher MVPA levels of 1200 MET‐min/week at 6‐month post‐diagnosis compared to our study (833.9 MET‐min/week). Others have also shown a similar level to ours (Littman et al. 552 MET‐min/week and Hong et al. 788 MET‐min/week) [14, 26, 44].

In context of national patterns, our data suggest that CBCS participants were more active than the general US population. Compared to the 2019 behavioral risk factor surveillance system and the 2020 national health interview survey, which reported that 23% and 24.2% of adults, respectively, engaged in sufficient MVPA, we found that 32% had sufficient MVPA at 6‐month post‐diagnosis [46, 47]. In previous studies of cancer survivors, ranges are wider and more in line with ours (32.0%–46.4%) [19, 26, 45, 47, 48]. Cancer survivors may experience increased health awareness leading to increases in PA, and rates of activity may differ by gender. We observed that the proportions of our study population with any MVPA increased relative to the baseline.

Considering correlates of MVPA, our data were concordant with Schilsky et al. which reported inverse associations between obesity and PA; however, those authors found no statistically significant association with race, education, and endocrine therapy [27]. Our findings that less aggressive disease was linked to any MVPA are consistent with previous studies showing that localized breast cancer, less aggressive treatment, and absence of lymphedema are associated with higher MVPA [17, 19, 20, 49]. However, a few previous studies have shown no associations with clinical correlates [25, 26, 49], and our results suggest that the effects of treatment may resolve (at least by 18‐month post‐diagnosis). This rebound aligns with Harrison et al. [26] which found that 60% of participants with no MVPA at 6‐ or 12‐month post‐diagnosis increased MVPA at 18‐month post‐diagnosis. Likewise, Littman et al. found frequency of any activity increased from 80% at 12‐month to 91.3% at 30‐month post‐diagnosis [14, 26]. One study found that a large fraction (40%) with high MVPA at baseline (on average 1.8 months post‐diagnosis) decreased their MVPA at 6‐month and reduced or maintained a low level of MVPA at 24‐month post‐diagnosis [20]. Some discrepancies may result from differences in baseline pre‐diagnosis MVPA levels, MVPA assessment methods (total MVPA vs. occupational and leisure‐time), or differences in distribution of cancer stage, treatment types, or SES of participants between cohorts [14, 23, 26, 50]; however, studies have observed variation over time.

Previous analyses of a subset of the CBCS3 by Hair et al. showed that Black women were more likely to reduce MVPA compared to White women, perhaps due to later‐stage diagnoses, a higher likelihood of receiving chemo and radiation therapy, and a lower likelihood of receiving endocrine therapy [19]. In this analysis, we added more participants and extended this analysis to later follow‐up, and found again that decreases and absence of MVPA post‐diagnosis were more common among Black women [6, 19, 27, 28, 44, 51]. The associations we found were similar in magnitude at 6‐month post‐diagnosis, but more precise, and we were able to show the positive trajectory at 18 months. We also emphasized any MVPA (rather than ‘sufficient MVPA’) in this analysis because several studies emphasize that any MVPA can have substantial benefits [4, 6]. By using this lower threshold, and accounting for individual‐ and community‐level socioeconomic factors, we contextualized the previous racial differences and were able to identify social correlates that may be better targets for intervention.

Strengths of this analysis included diverse participants due to oversampling of Black women and women under the age of 50. Few studies have evaluated MVPA up to or beyond 18‐month post‐diagnosis, and most of these studies included a small proportion of Black and younger women [14, 23, 26]. Compared to previous studies [27, 49, 52], our study included a larger percentage of participants who received chemotherapy and were diagnosed with cancer at later stages, which allowed us to estimate treatment‐related changes. We also included neighborhood factors, including census‐tract level SES variables, to elucidate social influences on MVPA among breast cancer survivors.

We also had some limitations. For example, we did not assess employment type. Occupational related PA may dilute our understanding of the benefits of behavioral change in MVPA among breast cancer survivors. We also note that we used self‐reported MVPA whereas many studies have begun using accelerometers; still, our results appear to be similar to those studies [14, 17, 20, 24, 25, 27, 49]. Recall bias is a concern inherent in studies that interview participants before, at the time of, and after diagnosis. The impact of distant recall of pre‐diagnosis MVPA on the validity of reported levels remains unclear. Recall bias may lead to an increased error in self‐reported PA levels, with individuals having lower baseline PA often over‐reporting their historical PA [53, 54]. Moreover, other studies found that recall differs by past activity type, where participants who performed vigorous PA tend to over‐report their past PA [53, 54]. Cancer survivors do seem to report higher MVPA levels than the general US population [19]. Despite the potential to overestimate PA, the concern remains that two‐thirds of breast cancer survivors in our study were below recommended PA guidelines.

We also note that 235 CBCS3 participants did not report MVPA at follow‐up. The absence of MVPA participation could cause selection bias if disease burden or adverse side effects and symptoms of cancer treatment (e.g., treatment‐related fatigue) influenced participants' MVPA and study participation.

### 4.1 Study Implications

This study underscores the relationship between MVPA and other social correlates (access to healthcare and community‐level SES). We found that participants with normal weight, income > $30K, some college‐level education, and low area deprivation reported MVPA at both 6‐ and 18‐month post‐diagnosis. Therefore, improving access to PA is an important consideration when designing interventions. For example, telehealth services (e.g., mobile health) for virtual care and personalized exercise counseling or programs may be needed to support those unable to attend in‐person [55, 56]. In one study, women with breast cancer who participated in mobile app‐based MVPA showed a significant increase in weekly steps [57, 58]. However, the appropriateness of an intervention may depend on the patient's needs, barriers to PA, and medical conditions prohibiting PA.

## 5 Conclusion

Among CBCS3 breast cancer survivors, participants with higher income and education, non‐Black race, higher social factors, and less aggressive treatment reported MVPA at both 6 and 18‐month post‐diagnosis. The more precise timing of MVPA resumption is also an important area of further study. Although studies have found that recommended levels of MVPA are associated with improved breast cancer survivorship [3, 9, 59], the timing and nature of interventions merit further consideration. Clinics should develop personalized MVPA programs tailored to each survivor's treatment stage and physical abilities. Providing support and regular follow‐up may help survivors resume PA and address individual barriers while integrating MVPA into survivorship care plans to ensure a more coordinated approach among health care providers and improve survivors recovery and quality of life. Understanding temporal and correlate patterns is one key to shaping survivorship care planning and supporting breast cancer survivors to attain recommended levels of MVPA.

## Author Contributions

**Michael A. Kebede:** conceptualization (lead), formal analysis (lead), investigation (lead), methodology (equal), software (equal), visualization (equal), writing – original draft (lead), writing – review and editing (equal). **Charles E. Matthews:** investigation (supporting), methodology (equal), writing – review and editing (equal). **Matthew R. Dunn:** formal analysis (supporting), methodology (supporting), visualization (equal), writing – review and editing (supporting). **Natasha R. Burse:** writing – review and editing (supporting). **Annie G. Howard:** formal analysis (equal), investigation (equal), methodology (equal), writing – review and editing (equal). **Kelly R. Evenson:** investigation (equal), methodology (lead), supervision (lead), visualization (equal), writing – original draft (equal), writing – review and editing (equal). **Melissa A. Troester:** conceptualization (equal), investigation (lead), methodology (lead), supervision (lead), visualization (lead), writing – review and editing (lead).

## Ethics Statement

The study was approved by the University of North Carolina Institutional Review Board in accordance with U.S. Common Rule. All study participants provided written informed consent prior to study entry. This study complied with relevant ethical regulations, including the Declaration of Helsinki.

## Conflicts of Interest

The authors declare no conflicts of interest.

## Supporting information


Tables S1–S16.

## Data Availability

The data analyzed in this study are taken from the Carolina Breast Cancer Study. However, these data are not publicly available. Investigators interested in accessing the data may submit a letter of intent for consideration.
