# Supplementary material for: Physical Activity Patterns According to Demographic, Social, and Clinical Correlates Among Breast Cancer Survivors
Source: Cancer Med. 2025 May 20;14(10):e70884. doi: 10.1002/cam4.70884 (PMC12092373; doi:10.1002/cam4.70884)
Supplement: Supplementary file 1 — Tables S1–S16. [file CAM4-14-e70884-s001.docx]

**Supplemental Tables**

**Supplemental Table 1.** Associations between demographic correlates and 6-month post-diagnosis and change in MVPA.^§^

|  | 6-month Post-Diagnosis MVPA | | | | | | | Change in MVPA | | | | | | |  |
| --- | --- | --- | --- | --- | --- | --- | --- | --- | --- | --- | --- | --- | --- | --- | --- |
|  | Sufficient  MVPA  N(%) | Insufficient MVPA  N (%) | Sufficient Vs No MVPA | p-value | No MVPA  N (%) | Insufficient Vs No MVPA | p-value | Increased  N (%) | No Change  N (%) | Increased Vs Decreased MVPA* | p-value | Decreased  N (%) | No change Vs Decreased MVPA * | p-value | |
| Race |  |  |  |  |  |  |  |  |  |  |  |  |  |  | |
| Black | 361 (12.07) | 343 (11.47) | 1. |  | 786 (26.28) | 1. |  | 206 (6.90) | 290 (9.71) | 1. |  | 992 (33.22) | 1. |  | |
| Non-Black | 595 (19.89) | 358 (11.97) | 2.04 | **<0.0001** | 548 (18.32) | 1.36 | **0.001** | 288 (9.65) | 273 (9.14) | 1.73 | **<0.0001** | 937 (31.38) | 1.39 | **0.007** | |
|  |  |  | (1.70, 2.45) |  |  | (1.13, 1.65) |  |  |  | (1.38, 2.17) |  |  | (1.09, 1.78) |  | |
| Age at diagnosis |  |  |  |  |  |  |  |  |  |  |  |  |  |  | |
| <50 | 469 (15.68) | 358 (11.97) | 1. |  | 660 (22.07) | 1. |  | 239 (8.00) | 248 (8.31) | 1. |  | 998 (33.42) | 1. |  | |
| ≥50 | 487 (16.28) | 343 (11.47) | 0.85 | 0.08 | 674 (22.53) | 0.85 | 0.09 | 255 (8.54) | 315 (10.55) | 1.01 | 0.89 | 931 (31.18) | 1.25 | 0.07 | |
|  |  |  | (0.71, 1.02) |  |  | (0.70, 1.03) |  |  |  | (0.81, 1.27) |  |  | (0.98, 1.60) |  | |
| Income |  |  |  |  |  |  |  |  |  |  |  |  |  |  | |
| ≤15K | 140 (4.68) | 137 (4.58) | 1. |  | 341 (11.40) | 1. |  | 82 (2.75) | 145 (4.86) | 1. |  | 388 (12.99) | 1. |  | |
| >15K-≤30K | 137 (4.58) | 123 (4.11) | 1.14 | 0.40 | 284 (9.50) | 1.06 | 0.69 | 75 (2.51) | 104 (3.48) | 1.00 | 0.98 | 364 (12.19) | 0.83 | 0.33 | |
|  |  |  | (0.84, 1.53) |  |  | (0.79, 1.42) |  |  |  | (0.68, 1.45) |  |  | (0.57, 1.21) |  | |
| >30K | 679 (22.70) | 441 (14.74) | 2.10 | **<0.0001** | 709 (23.70) | 1.45 | **0.002** | 337 (11.29) | 314 (10.52) | 1.72 | **0.0004** | 1177 (39.42) | 1.12 | 0.46 | |
|  |  |  | (1.66, 2.67) |  |  | (1.15, 1.84) |  |  |  | (1.27, 2.32) |  |  | (0.83, 1.51) |  | |
| Education |  |  |  |  |  |  |  |  |  |  |  |  |  |  | |
| <= HS Graduate | 475 (15.88) | 426 (14.24) | 1. |  | 911 (30.46) | 1. |  | 269 (9.01) | 366 (12.26) | 1. |  | 1172 (39.25) | 1. |  | |
| >= Some college | 481 (16.08) | 275 (9.19) | 2.05 | **<0.0001** | 423(14.14) | 1.34 | **0.004** | 225 (7.54) | 197 (6.60) | 1.64 | **<0.0001** | 757 (25.35) | 1.21 | 0.12 | |
|  |  |  | (1.70, 2.46) |  |  | (1.10, 1.62) |  |  |  | (1.31, 2.06) |  |  | (0.95, 1.55) |  | |

§Analysis using multivariable-adjusted polytomous model to calculate odds ratios and 95% CI (N=2994).

Abbreviations: CI= confidence interval; MVPA= moderate to vigorous physical activity; HS= high school

a. Multivariable adjusted model includes adjustment for minutes per week of physical activity pre-diagnosis, age at diagnosis, endocrine therapy, lymphedema, stage, and chemotherapy.

b. To evaluate changes between pre-diagnosis and 6-month post-diagnosis MVPA, the categorical variable is defined as follows: **i**ncreased activity by > 30 minutes; decreased activity by > 30 minutes; no change, within 30 minutes.

**Supplemental Table 2.** Social level correlates associated with 6-month post-diagnosis and change in MVPA.^§^

|  | 6-month Post-Diagnosis MVPA | | | | | | |  | Change in MVPA^*^ | | | | | |
| --- | --- | --- | --- | --- | --- | --- | --- | --- | --- | --- | --- | --- | --- | --- |
|  | Sufficient  MVPA  N(%) | Insufficient MVPA  N (%) | Sufficient Vs No MVPA | p-value | No MVPA  N (%) | Insufficient Vs No MVPA | p-value | Increased  N (%) | No Change  N (%) | Increased Vs Decreased MVPA* | p-value | Decreased  N (%) | No change Vs Decreased MVPA * | p-value |
| Access to healthcare |  |  |  |  |  |  |  |  |  |  |  |  |  |  |
| More barriers | 183 (6.12) | 163 (5.45) | 1. |  | 345 (11.53) | 1. |  | 100 (3.35) | 146 (4.89) | 1. |  | 443 (14.84) | 1. |  |
| Fewer barriers | 773 (25.84) | 538 (18.00) | 1.27 | **0.03** | 989 (33.07) | 1.08 | 0.50 | 394 (13.19) | 417 (13.97) | 1.34 | **0.03** | 1486 (49.77) | 1.12 | 0.43 |
|  |  |  | (1.02, 1.57) |  |  | (0.87, 1.34) |  |  |  | (1.02, 1.75) |  |  | (0.85, 1.47) |  |
| Community level SES |  |  |  |  |  |  |  |  |  |  |  |  |  |  |
| Deprivation |  |  |  |  |  |  |  |  |  |  |  |  |  |  |
| High | 298 (9.96) | 267 (8.93) | 1. |  | 597 (19.96) | 1. |  | 159 (5.32) | 233 (7.80) | 1. |  | 767 (25.69) | 1. |  |
| Low | 658 (22.00) | 434 (14.51) | 1.66 | **<0.0001** | 737 (24.64) | 1.25 | **0.02** | 335 (11.22) | 330 (11.05) | 1.66 | **<0.0001** | 1162 (38.91) | 1.29 | **0.05** |
|  |  |  | (1.38, 2.00) |  |  | (1.04, 1.51) |  |  |  | (1.31, 2.10) |  |  | (0.99, 1.67) |  |
| Assets |  |  |  |  |  |  |  |  |  |  |  |  |  |  |
| Low | 470 (15.71) | 382 (12.77) | 1. |  | 850 (28.42) | 1. |  | 247 (8.27) | 337 (11.29) | 1. |  | 1114 (37.31) | 1. |  |
| High | 486 (16.25) | 319 (10.67) | 1.67 | **<0.0001** | 484 (16.18) | 1.42 | **0.0003** | 247 (8.27) | 226 (7.57) | 1.64 | **<0.0001** | 815 (27.29) | 1.25 | 0.07 |
|  |  |  | (1.40, 2.00) |  |  | (1.17, 1.71) |  |  |  | (1.32, 2.04) |  |  | (0.98, 1.59) |  |

§Analysis using multivariable-adjusted polytomous model to calculate odds ratios and 95% CI (N=2994).

Abbreviations: CI= confidence interval; MVPA= moderate to vigorous physical activity; SES = socioeconomic status.

a. Multivariable adjusted model includes adjustment for minutes per week of physical activity pre-diagnosis, age at diagnosis, endocrine therapy, lymphedema, stage, and chemotherapy.

b. To evaluate changes between pre-diagnosis and 6-month post-diagnosis MVPA, the categorical variable is defined as follows: increased activity by > 30 minutes; decreased activity by > 30 minutes; no change, within 30 minutes.

c. Access to healthcare (fewer vs more barriers) was defined by insurance, urban/rural residence, job loss, and transportation issues.

d. Deprivation includes no vehicle, public assistance, unemployment, crowded housing, renting, single householder, female householder, and below poverty line whereas assets include Male/female professionals.

**Supplemental Table 3.** Clinical correlates associated with 6-month post-diagnosis and change in MVPA.^§^

|  | 6-month Post-Diagnosis MVPA | | | | | | |  | Change in MVPA^*^ | | | | | |
| --- | --- | --- | --- | --- | --- | --- | --- | --- | --- | --- | --- | --- | --- | --- |
|  | Sufficient  MVPA  N (%) | Insufficient MVPA  N (%) | Sufficient Vs No MVPA | p-value | No MVPA  N (%) | Insufficient Vs No MVPA | p-value | Increased  N (%) | No Change  N (%) | Increased Vs Decreased MVPA* | p-value | Decreased  N (%) | No change Vs Decreased MVPA * | p-value |
| BMI |  |  |  |  |  |  |  |  |  |  |  |  |  |  |
| ≥30 | 364 (12.22) | 345 (11.58) | 1. |  | 725 (24.34) | 1. |  | 219 (7.36) | 292 (9.82) | 1. |  | 919 (30.90) | 1. |  |
| 25 - <30 | 276 (9.26) | 187 (6.28) | 1.45 | **0.0006** | 357 (11.98) | 1.08 | 0.51 | 139 (4.67) | 153 (5.14) | 1.28 | 0.06 | 528 (17.75) | 1.20 | 0.20 |
|  |  |  | (1.17, 1.80) |  |  | (0.86, 1.34) |  |  |  | (0.99, 1.67) |  |  | (0.91, 1.59) |  |
| <25 | 309 (10.37) | 169 (5.67) | 2.02 | **<0.0001** | 247 (8.29) | 1.31 | **0.02** | 133 (4.47) | 116 (3.90) | 1.77 | **<0.0001** | 475 (15.97) | 1.58 | **0.003** |
|  |  |  | (1.62, 2.52) |  |  | (1.03, 1.67) |  |  |  | (1.35, 2.32) |  |  | (1.16, 2.15) |  |
| Endocrine therapy |  |  |  |  |  |  |  |  |  |  |  |  |  |  |
| No | 248 (8.44) | 202 (6.88) | 1. |  | 506 (17.23) | 1. |  | 125 (4.26) | 175 (5.97) | 1. |  | 661 (22.3) | 1. |  |
| Yes | 692 (23.56) | 484 (16.48) | 1.54 | **<0.0001** | 805 (27.41) | 1.44 | **0.0005** | 355 (12.11) | 378 (12.89) | 1.35 | **0.02** | 1255 (42.3) | 1.10 | 0.48 |
|  |  |  | (1.26, 1.88) |  |  | (1.17, 1.77) |  |  |  | (1.05, 1.74) |  |  | (0.84, 1.43) |  |
| Chemotherapy |  |  |  |  |  |  |  |  |  |  |  |  |  |  |
| Yes | 494 (16.82) | 428 (14.57) | 1. |  | 910 (30.98) | 1. |  | 262 (8.94) | 318 (10.85) | 1. |  | 1249 (42.60) | 1. |  |
| No | 446 (15.19) | 258 (8.78) | 1.66 | **<0.0001** | 401 (13.65) | 1.18 | 0.16 | 218 (7.44) | 235 (8.02) | 1.71 | **0.0001** | 650 (22.17) | 1.86 | **<0.0001** |
|  |  |  | (1.33, 2.07) |  |  | (0.93, 1.50) |  |  |  | (1.30, 2.24) |  |  | (1.38, 2.50) |  |
| Lymphedema |  |  |  |  |  |  |  |  |  |  |  |  |  |  |
| Yes | 36 (1.23) | 43 (1.46) | 1. |  | 92 (3.13) | 1. |  | 25 (0.85) | 32 (1.09) | 1. |  | 114 (3.89) | 1. |  |
| No | 904 (30.79) | 643 (21.90) | 1.56 | **0.03** | 1218 (41.49) | 1.06 | 0.78 | 455 (15.52) | 520 (17.74) | 1.24 | 0.39 | 1785 (60.90) | 1.28 | 0.35 |
|  |  |  | (1.03, 2.36) |  |  | (0.72, 1.54) |  |  |  | (0.75, 2.04) |  |  | (0.76, 2.14) |  |
| Stage |  |  |  |  |  |  |  |  |  |  |  |  |  |  |
| 3 or 4 | 118 (4.02) | 123 (4.19) | 1. |  | 255 (8.68) | 1. |  | 65 (2.22) | 101 (3.44) | 1. |  | 328 (11.19) | 1. |  |
| 1 | 467(15.90) | 295 (10.04) | 1.54 | **0.004** | 459 (15.63) | 1.21 | 0.20 | 227 (7.74) | 230 (7.84) | 1.22 | 0.29 | 763 (26.02) | 0.81 | 0.28 |
|  |  |  | (1.15, 2.07) |  |  | (0.90, 1.62) |  |  |  | (0.84, 1.76) |  |  | (0.55, 1.18) |  |
| 2 | 357 (12.09) | 268 (9.12) | 1.19 | 0.20 | 597 (20.33) | 0.92 | 0.54 | 188 (6.41) | 222 (7.57) | 1.09 | 0.63 | 808 (27.56) | 0.81 | 0.22 |
|  |  |  | (0.91, 1.55) |  |  | (0.71, 1.20) |  |  |  | (0.77, 1.52) |  |  | (0.57, 1.14) |  |

§Analysis using multivariable-adjusted polytomous model to calculate odds ratios and 95% CI (N=2940).

Abbreviations: CI= confidence interval; MVPA= moderate to vigorous physical activity; BMI=body mass index;

a. Multivariable adjusted model includes adjustment for minutes per week of physical activity pre-diagnosis, age at diagnosis, endocrine therapy, lymphedema, stage, and chemotherapy.

b. To evaluate changes between pre-diagnosis and 6-month post-diagnosis MVPA, the categorical variable is defined as follows: increased activity by > 30 minutes; decreased activity by > 30 minutes; no change, within 30 minutes.

c. Participants who did not receive surgery were excluded (n=54).

**Supplemental Table 4.** Demographic correlates associated with 6-month post-diagnosis and change in MVPA overall and by race.^§^

|  | Non-Black N=1502) | | Black (N=1492) | | Overall (N=2994) | |
| --- | --- | --- | --- | --- | --- | --- |
|  | **6-month Post-diagnosis** | **Change in MVPA** | **6-month Post-diagnosis** | **Change in MVPA** | **6-month Post-diagnosis** | **Change in MVPA** |
| Age at diagnosis |  |  |  |  |  |  |
| <50 | 2030.9(1745.0, 2316.7) | -3059.4(-3345.2, -2773.5) | 900.5(690.8, 1110.2) | -3516.7(-3726.5, -3307.0) | 1779.5(1584.3, 1974.7) | -3703.7(-3898.9, -3508.6) |
| ≥50 | 1991.9(1711.0, 2272.9) | -3098.3(-3379.3, -2817.4) | 978.2(765.6, 1190.8) | -3439.0(-3651.6, -3226.4) | 1773.1(1578.1, 1968.0) | -3710.1(-3905.1, -3515.2) |
| Income |  |  |  |  |  |  |
| >30K | 2093.8(1814.6,2373.0) | -2996.5(-3275.7, -2717.3) | 1058.4(841.7,1275.1) | -3358.9(-3575.6, -3142.2) | 1917.4(1723.1, 2111.7) | -3565.9(-3760.2, -3371.5) |
| >15K-≤30K | 1972.9(1626.3,2319.5) | -3117.3(-3463.9, -2770.7) | 822.7(589.8, 1055.6) | -3594.5(-3827.5, -3361.6) | 1624.4(1406.2,1842.5) | -3858.9(-4077.0, -3640.7) |
| ≤15K | 1768.6(1423.6,2113.5) | -3321.7(-3666.6, -2976.8) | 821.8(594.6, 1049.0) | -3595.4(-3822.6, -3368.2) | 1561.1(1346.7,1775.5) | -3922.1(-4136.5, -3707.7) |
| Education |  |  |  |  |  |  |
| ≥ Some college | 2092.0(1804.9, 2379.2) | -2998.2(-3285.4, -2711.0) | 972.1(750.3, 1193.9) | -3445.1(-3667.0, -3223.3) | 1871.0(1671.2, 2070.9) | -3612.2(-3812.0, -3412.4) |
| ≤HS Education | 1953.8(1671.8, 2235.8) | -3136.5(-3418.4, -2854.5) | 892.4(681.2, 1103.7) | -3524.8(-3736.1, -3313.6) | 1699.7(1505.6,1893.7) | -3783.5(-3977.6, -3589.5) |

§Evaluated through Met-minutes/week to derive multivariable adjusted mean and 95%CI.

Abbreviations: CI= confidence interval; MVPA= moderate to vigorous physical activity; HS= high school.

1. Multivariable adjusted model includes adjustment for Met-minutes/week of physical activity pre-diagnosis, age at diagnosis, endocrine therapy, lymphedema, stage, and chemotherapy.
2. Change in MVPA is subtraction of 6-month mean met-minutes of MVPA from pre-diagnosis MVPA.

**Supplemental Table 5.** Social level correlates associated with 6-month post-diagnosis and change in MVPA overall and by race.^§^

|  | Non-Black (N=1502) | | Black (N=1492) | | Overall (N=2994) | |
| --- | --- | --- | --- | --- | --- | --- |
|  | **6-month Post-diagnosis** | **Change in MVPA** | **6-month Post-diagnosis** | **Change in MVPA** | **6-month Post-diagnosis** | **Change in MVPA** |
| Access to healthcare |  |  |  |  |  |  |
| Fewer barriers | 2006.3(1733.2, 2279.3) | -3084.0(-3357.0, -2811.0) | 953.2(746.3, 1160.1) | -3464.0(-3670.9, -3257.2) | 1782.0(1591.6, 1972.3) | -3701.3(-3891.6, -3510.9) |
| More barriers | 2072.7(1744.6, 2400.9) | -3017.5(-3345.7, -2689.4) | 849.4(619.5, 1079.3) | -3567.9(-3797.8, -3338.0) | 1727.7(1512.8, 1942.6) | -3755.5(-3970.4, -3540.6) |
| Community level SES |  |  |  |  |  |  |
| Deprivation |  |  |  |  |  |  |
| Low | 2066.0(1791.8, 2340.3) | -3024.2(-3298.5, -2750.0) | 993.9(775.0, 1212.8) | -3423.3(-3642.2, -3204.4) | 1859.9(1666.2,2053.7) | -3623.3(-3817.0, -3429.6) |
| High | 1827.5(1509.8, 2145.3) | -3262.7(-3580.5, -2945.0) | 879.5(670.1, 1088.9) | -3537.8(-3747.2, -3328.4) | 1645.5(1446.3,1844.7) | -3837.7(-4036.9, -3638.6) |
| Assets |  |  |  |  |  |  |
| high | 2082.2(1802.4, 2362.0) | -3008.1(-3287.9, -2728.3) | 986.9(761.2, 1212.5) | -3430.4(-3656.1, -3204.7) | 1865.3(1667.3,2063.3) | -3618.0(-3816.0, -3420.0) |
| low | 1921.3(1632.3, 2210.3) | -3169.0(-3458.0, -2880.0) | 894.2(686.1, 1102.3) | -3523.0(-3731.1, -3314.9) | 1694.0(1499.4, 1888.7) | -3789.2(-3983.8, -3594.5) |

§Evaluated through Met-minutes/week to derive multivariable-adjusted mean and 95%CI.

Abbreviations: CI= confidence interval; MVPA= moderate to vigorous physical activity; SES=socioeconomic status.

a. Multivariable adjusted model includes adjustment for Met-minutes/week of physical activity pre-diagnosis, age at diagnosis, endocrine therapy, lymphedema, stage, and chemotherapy.

b. Access to healthcare (fewer vs more barriers) was defined by insurance, urban/rural residence, job loss, and transportation issues.

c. Deprivation includes no vehicle, public assistance, unemployed, crowded housing, renting, single householder, female householder, and below poverty line whereas assets include Male/female professional.

d. Change in MVPA is subtraction of 6-month mean met-minutes of MVPA from pre-diagnosis MVPA.

**Supplemental Table 6.** Clinical correlates associated with 6-month post-diagnosis and change in MVPA overall and by race.^§^

|  | | Non-Black (N=1485) | | | | Black (N=1454) | | | | Overall (N=2940) | |
| --- | --- | --- | --- | --- | --- | --- | --- | --- | --- | --- | --- |
|  | | **6-month Post-diagnosis** | | **Change in MVPA** | | **6-month Post-diagnosis** | | **Change in MVPA** | | **6-month Post-diagnosis** | **Change in MVPA** |
| BMI | |  | |  | |  | |  | |  |  |
| <25 | | 2213.5(1918.3, 2508.7) | | -2876.7(-3171.9, -2581.5) | | 993.7(722.6,1264.9) | | -3423.5(-3694.7, -3152.4) | | 2024.6(1811.9, 2237.3) | -3458.6(-3671.3, -3246.0) |
| 25 - <30 | | 1941.2(1638.1, 2244.2) | | -3149.1(-3452.1, -2846.1) | | 1018.8(784.3,1253.3) | | -3398.4(-3633.0, -3163.9) | | 1804.3(1596.2,2012.4) | -3678.9(-3887.0, -3470.9) |
| ≥30 | | 1874.1(1581.2,2167.1) | | -3216.1(-3509.0, -2923.2) | | 876.8(668.3,1085.3) | | -3540.4(-3748.9, -3331.9) | | 1630.8(1435.0,1826.6) | -3852.4(-4048.2, -3656.6) |
| Endocrine therapy | |  | |  | |  | |  | |  |  |
| Yes | | 2038.2(1772.1, 2304.3) | | -3052.1(-3318.1, -2786.0) | | 1005.5(797.3, 1213.7) | | -3411.8(-3620.0, -3203.6) | | 1839.8(1650.9, 2028.7) | -3643.4(-3832.4, -3454.5) |
| No | | 1994.0(1684.3, 2303.8) | | -3096.2(-3405.9, -2786.5) | | 843.0(622.0, 1063.9) | | -3574.3(-3795.3, -3353.3) | | 1701.5(1495.3, 1907.7) | -3781.7(-3987.9, -3575.5) |
| Chemotherapy | |  | |  | |  | |  | |  |  |
| yes | | 1866.2(1592.6, 2139.9) | | -3224.0(-3497.7, -2950.4) | | 776.6(570.5, 982.7) | | -3640.6(-3846.7, -3434.5) | | 1607.6(1417.3, 1797.8) | -3875.7(-4065.9, -3685.5) |
| no | | 2166.0(1858.7, 2473.3) | | -2924.2(-3231.5, -2617.0) | | 1071.8(837.9, 1305.7) | | -3345.5(-3579.4, -3111.5) | | 1933.7(1723.2, 2144.2) | -3549.5(-3760.0, -3339.0) |
| Lymphedema |  | |  | |  | |  | |  | |  |
| No | 2105.3(1906.0, 2304.5) | | -2985.0(-3184.2, -2785.7) | | 986.2(815.5, 1157.0) | | -3431.1(-3601.8, -3260.3) | | 1857.8(1700.1, 2015.6) | | -3625.4(-3783.1, -3467.6) |
| Yes | 1927.0(1497.9, 2356.0) | | -3163.3(-3592.3, -2734.2) | | 862.2(564.2, 1160.2) | | -3555.0(-3853.0, -3257.1) | | 1683.4(1413.1, 1953.8) | | -3799.8(-4070.1, -3529.4) |
| Stage |  | |  | |  | |  | |  | |  |
| 1 | 2126.8(1842.8, 2410.7) | | -2963.5(-3247.4, -2679.5) | | 944.8(727.4, 1162.2) | | -3472.5(-3689.9, -3255.1) | | 1867.5(1670.0, 2065.0) | | -3615.7(-3813.2, -3418.2) |
| 2 | 1960.5(1672.1, 2248.9) | | -3129.7(-3418.2, -2841.3) | | 943.5(723.7, 1163.3) | | -3473.8(-3693.6, -3254.0) | | 1738.9(1538.7, 1939.1) | | -3744.4(-3944.5, -3544.2) |
| 3 or 4 | 1961.1(1616.2, 2305.9) | | -3129.2(-3474.1, -2784.3) | | 884.4(636.0, 1132.7) | | -3532.9(-3781.2, -3284.5) | | 1705.5(1479.1, 1932.0) | | -3777.7(-4004.1, -3551.2) |

§ Evaluated through Met-minutes/week to derive multivariable-adjusted mean and 95%CI.

Abbreviations: CI= confidence interval; MVPA= moderate to vigorous physical activity; BMI=body mass index;

1. Multivariable adjusted model includes adjustment for Met-minutes/week of physical activity pre-diagnosis, age at diagnosis, endocrine therapy, lymphedema, stage, and chemotherapy.
2. Participants who did not receive surgery were excluded (n=54).
3. Change in MVPA is subtraction of 6-month mean met-minutes of MVPA from pre-diagnosis MVPA.

**Supplemental Table 7.** Associations between demographic correlates and 6-month post-diagnosis and change in MVPA.^§^

|  | 6-month Post-Diagnosis MVPA | | | | | | | Change in MVPA | | | | | | |  |
| --- | --- | --- | --- | --- | --- | --- | --- | --- | --- | --- | --- | --- | --- | --- | --- |
|  | Sufficient  MVPA  N(%) | Insufficient MVPA  N (%) | Sufficient Vs No MVPA | p-value | No MVPA  N (%) | Insufficient Vs No MVPA | p-value | Increased  N (%) | No Change  N (%) | Increased Vs Decreased MVPA* | p-value | Decreased  N (%) | No change Vs Decreased MVPA * | p-value | |
| Race |  |  |  |  |  |  |  |  |  |  |  |  |  |  | |
| Black | 361 (12.07) | 343 (11.47) | 1. |  | 786 (26.28) | 1. |  | 206 (6.90) | 290 (9.71) | 1. |  | 992 (33.22) | 1. |  | |
| Non-Black | 595 (19.89) | 358 (11.97) | 1.62 | **<0.0001** | 548 (18.32) | 1.29 | **0.01** | 288 (9.65) | 273 (9.14) | 1.44 | **0.002** | 937 (31.38) | 1.26 | **0.04** | |
|  |  |  | (1.33, 1.98) |  |  | (1.05, 1.59) |  |  |  | (1.14, 1.83) |  |  | (1.01, 1.58) |  | |
| Age at diagnosis |  |  |  |  |  |  |  |  |  |  |  |  |  |  | |
| <50 | 469 (15.68) | 358 (11.97) | 1. |  | 660 (22.07) | 1. |  | 239 (8.00) | 248 (8.31) | 1. |  | 998 (33.42) | 1. |  | |
| ≥50 | 487 (16.28) | 343 (11.47) | 1.23 | **0.02** | 674 (22.53) | 1.02 | 0.88 | 255 (8.54) | 315 (10.55) | 1.24 | **0.04** | 931 (31.18) | 1.31 | **0.009** | |
|  |  |  | (1.03, 1.48) |  |  | (0.84, 1.23) |  |  |  | (1.01, 1.53) |  |  | (1.07, 1.60) |  | |
| Income |  |  |  |  |  |  |  |  |  |  |  |  |  |  | |
| ≤15K | 140 (4.68) | 137 (4.58) | 1. |  | 341 (11.40) | 1. |  | 82 (2.75) | 145 (4.86) | 1. |  | 388 (12.99) | 1. |  | |
| >15K-≤30K | 137 (4.58) | 123 (4.11) | 1.18 | 0.28 | 284 (9.50) | 1.09 | 0.56 | 75 (2.51) | 104 (3.48) | 0.97 | 0.89 | 364 (12.19) | 0.80 | 0.16 | |
|  |  |  | (0.87, 1.60) |  |  | (0.81, 1.46) |  |  |  | (0.68, 1.39) |  |  | (0.59, 1.09) |  | |
| >30K | 679 (22.70) | 441 (14.74) | 1.78 | **<0.0001** | 709 (23.70) | 1.37 | **0.01** | 337 (11.29) | 314 (10.52) | 1.22 | 0.19 | 1177 (39.42) | 0.82 | 0.15 | |
|  |  |  | (1.38, 2.30) |  |  | (1.07, 1.77) |  |  |  | (0.90, 1.65) |  |  | (0.63, 1.07) |  | |
| Education |  |  |  |  |  |  |  |  |  |  |  |  |  |  | |
| <= HS Graduate | 475 (15.88) | 426 (14.24) | 1. |  | 911 (30.46) | 1. |  | 269 (9.01) | 366 (12.26) | 1. |  | 1172 (39.25) | 1. |  | |
| >= Some college | 481 (16.08) | 275 (9.19) | 1.59 | **<0.0001** | 423(14.14) | 1.13 | 0.25 | 225 (7.54) | 197 (6.60) | 1.22 | 0.09 | 757 (25.35) | 1.02 | 0.85 | |
|  |  |  | (1.30, 1.94) |  |  | (0.91, 1.40) |  |  |  | (0.97, 1.54) |  |  | (0.81, 1.29) |  | |

§Analysis using multivariable-adjusted polytomous model to calculate odds ratios and 95% CI (N=2994).

Abbreviations: CI= confidence interval; MVPA= moderate to vigorous physical activity; HS= high school

a. Multivariable adjusted model includes adjustment for age at diagnosis, Met-minutes/week of physical activity pre-diagnosis, income, education, bmi, community level SES (deprivation, assets), and access to care.

b. To evaluate changes between pre-diagnosis and 6-month post-diagnosis MVPA, the categorical variable is defined as follows: increased activity by > 30 minutes; decreased activity by > 30 minutes; no change, within 30 minutes.

**Supplemental Table 8.** Social level correlates associated with 6-month post-diagnosis and change in MVPA.^§^

|  | 6-month Post-Diagnosis MVPA | | | | | | |  | Change in MVPA^*^ | | | | | |
| --- | --- | --- | --- | --- | --- | --- | --- | --- | --- | --- | --- | --- | --- | --- |
|  | Sufficient  MVPA  N(%) | Insufficient MVPA  N (%) | Sufficient Vs No MVPA | p-value | No MVPA  N (%) | Insufficient Vs No MVPA | p-value | Increased  N (%) | No Change  N (%) | Increased Vs Decreased MVPA* | p-value | Decreased  N (%) | No change Vs Decreased MVPA * | p-value |
| Access to healthcare |  |  |  |  |  |  |  |  |  |  |  |  |  |  |
| More barriers | 183 (6.12) | 163 (5.45) | 1. |  | 345 (11.53) | 1. |  | 100 (3.35) | 146 (4.89) | 1. |  | 443 (14.84) | 1. |  |
| Fewer barriers | 773 (25.84) | 538 (18.00) | 1.04 | 0.75 | 989 (33.07) | 0.94 | 0.63 | 394 (13.19) | 417 (13.97) | 0.98 | 0.86 | 1486 (49.77) | 0.90 | 0.40 |
|  |  |  | (0.83, 1.30) |  |  | (0.75, 1.19) |  |  |  | (0.75, 1.28) |  |  | (0.70, 1.15) |  |
| Community level SES |  |  |  |  |  |  |  |  |  |  |  |  |  |  |
| Deprivation |  |  |  |  |  |  |  |  |  |  |  |  |  |  |
| High | 298 (9.96) | 267 (8.93) | 1. |  | 597 (19.96) | 1. |  | 159 (5.32) | 233 (7.80) | 1. |  | 767 (25.69) | 1. |  |
| Low | 658 (22.00) | 434 (14.51) | 1.20 | 0.12 | 737 (24.64) | 0.99 | 0.92 | 335 (11.22) | 330 (11.05) | 1.24 | 0.13 | 1162 (38.91) | 1.06 | 0.66 |
|  |  |  | (0.95, 1.52) |  |  | (0.77, 1.26) |  |  |  | (0.94, 1.64) |  |  | (0.82, 1.38) |  |
| Assets |  |  |  |  |  |  |  |  |  |  |  |  |  |  |
| Low | 470 (15.71) | 382 (12.77) | 1. |  | 850 (28.42) | 1. |  | 247 (8.27) | 337 (11.29) | 1. |  | 1114 (37.31) | 1. |  |
| High | 486 (16.25) | 319 (10.67) | 1.12 | 0.32 | 484 (16.18) | 1.31 | **0.03** | 247 (8.27) | 226 (7.57) | 1.17 | 0.25 | 815 (27.29) | 1.06 | 0.66 |
|  |  |  | (0.89, 1.42) |  |  | (1.02, 1.68) |  |  |  | (0.89, 1.55) |  |  | (0.81, 1.39) |  |

§Analysis using multivariable-adjusted polytomous model to calculate odds ratios and 95% CI (N=2994).

Abbreviations: CI= confidence interval; MVPA= moderate to vigorous physical activity; SES = socioeconomic status.

a. Multivariable adjusted model includes adjustment for age at diagnosis, Met-minutes/week of physical activity pre-diagnosis, income, education, bmi, community level SES (deprivation, assets), and access to care.

b. To evaluate changes between pre-diagnosis and 6-month post-diagnosis MVPA, the categorical variable is defined as follows: increased activity by > 30 minutes; decreased activity by > 30 minutes; no change, within 30 minutes.

c. Access to healthcare (fewer vs more barriers) was defined by insurance, urban/rural residence, job loss, and transportation issues.

d. Deprivation includes no vehicle, public assistance, unemployment, crowded housing, renting, single householder, female householder, and below poverty line whereas assets include Male/female professionals.

**Supplemental Table 9.** Clinical correlates associated with 6-month post-diagnosis and change in MVPA.^§^

|  | 6-month Post-Diagnosis MVPA | | | | | | |  | Change in MVPA^*^ | | | | | |
| --- | --- | --- | --- | --- | --- | --- | --- | --- | --- | --- | --- | --- | --- | --- |
|  | Sufficient  MVPA  N (%) | Insufficient MVPA  N (%) | Sufficient Vs No MVPA | p-value | No MVPA  N (%) | Insufficient Vs No MVPA | p-value | Increased  N (%) | No Change  N (%) | Increased Vs Decreased MVPA* | p-value | Decreased  N (%) | No change Vs Decreased MVPA * | p-value |
| BMI |  |  |  |  |  |  |  |  |  |  |  |  |  |  |
| ≥30 | 364 (12.22) | 345 (11.58) | 1. |  | 725 (24.34) | 1. |  | 219 (7.36) | 292 (9.82) | 1. |  | 919 (30.90) | 1. |  |
| 25 - <30 | 276 (9.26) | 187 (6.28) | 1.29 | **0.02** | 357 (11.98) | 1.03 | 0.82 | 139 (4.67) | 153 (5.14) | 1.10 | 0.44 | 528 (17.75) | 1.00 | 0.96 |
|  |  |  | (1.04, 1.60) |  |  | (0.82,1.28) |  |  |  | (0.86, 1.41) |  |  | (0.79, 1.27) |  |
| <25 | 309 (10.37) | 169 (5.67) | 1.84 | **<0.0001** | 247 (8.29) | 1.28 | **0.05** | 133 (4.47) | 116 (3.90) | 1.30 | **0.05** | 475 (15.97) | 1.06 | 0.64 |
|  |  |  | (1.47, 2.31) |  |  | (1.00, 1.63) |  |  |  | (1.00, 1.69) |  |  | (0.82, 1.38) |  |
| Endocrine therapy |  |  |  |  |  |  |  |  |  |  |  |  |  |  |
| No | 248 (8.44) | 202 (6.88) | 1. |  | 506 (17.23) | 1. |  | 125 (4.26) | 175 (5.97) | 1. |  | 661 (22.3) | 1. |  |
| Yes | 692 (23.56) | 484 (16.48) | 1.62 | **<0.0001** | 805 (27.41) | 1.46 | **0.0002** | 355 (12.11) | 378 (12.89) | 1.40 | **0.004** | 1255 (42.3) | 1.10 | 0.36 |
|  |  |  | (1.34, 1.97) |  |  | (1.20, 1.78) |  |  |  | (1.11, 1.76) |  |  | (0.89, 1.37) |  |
| Chemotherapy |  |  |  |  |  |  |  |  |  |  |  |  |  |  |
| Yes | 494 (16.82) | 428 (14.57) | 1. |  | 910 (30.98) | 1. |  | 262 (8.94) | 318 (10.85) | 1. |  | 1249 (42.60) | 1. |  |
| No | 446 (15.19) | 258 (8.78) | 2.13 | **<0.0001** | 401 (13.65) | 1.46 | **0.0002** | 218 (7.44) | 235 (8.02) | 1.70 | **<0.0001** | 650 (22.17) | 1.45 | **0.0005** |
|  |  |  | (1.76, 2.57) |  |  | (1.19, 1.78) |  |  |  | (1.37, 2.12) |  |  | (1.18, 1.80) |  |
| Lymphedema |  |  |  |  |  |  |  |  |  |  |  |  |  |  |
| Yes | 36 (1.23) | 43 (1.46) | 1. |  | 92 (3.13) | 1. |  | 25 (0.85) | 32 (1.09) | 1. |  | 114 (3.89) | 1. |  |
| No | 904 (30.79) | 643 (21.90) | 1.57 | **0.03** | 1218 (41.49) | 1.07 | 0.71 | 455 (15.52) | 520 (17.74) | 1.17 | 0.50 | 1785 (60.90) | 1.16 | 0.48 |
|  |  |  | (1.04, 2.37) |  |  | (0.74, 1.56) |  |  |  | (0.74, 1.83) |  |  | (0.76, 1.77) |  |
| Stage |  |  |  |  |  |  |  |  |  |  |  |  |  |  |
| 3 or 4 | 118 (4.02) | 123 (4.19) | 1. |  | 255 (8.68) | 1. |  | 65 (2.22) | 101 (3.44) | 1. |  | 328 (11.19) | 1. |  |
| 1 | 467(15.90) | 295 (10.04) | 1.83 | **<0.0001** | 459 (15.63) | 1.26 | 0.08 | 227 (7.74) | 230 (7.84) | 1.32 | 0.07 | 763 (26.02) | 1.00 | 0.99 |
|  |  |  | (1.41, 2.38) |  |  | (0.97, 1.63) |  |  |  | (0.97, 1.78) |  |  | (0.75, 1.32) |  |
| 2 | 357 (12.09) | 268 (9.12) | 1.18 | 0.21 | 597 (20.33) | 0.89 | 0.38 | 188 (6.41) | 222 (7.57) | 1.01 | 0.93 | 808 (27.56) | 0.86 | 0.30 |
|  |  |  | (0.91, 1.53) |  |  | (0.69, 1.15) |  |  |  | (0.75, 1.37) |  |  | (0.65, 1.14) |  |

§Analysis using multivariable-adjusted polytomous model to calculate odds ratios and 95% CI (N=2940).

Abbreviations: CI= confidence interval; MVPA= moderate to vigorous physical activity; BMI=body mass index;

a. Multivariable adjusted model includes adjustment for age at diagnosis, Met-minutes/week of physical activity pre-diagnosis, income, education, bmi, community level SES (deprivation, assets), and access to care.

b. To evaluate changes between pre-diagnosis and 6-month post-diagnosis MVPA, the categorical variable is defined as follows: increased activity by > 30 minutes; decreased activity by > 30 minutes; no change, within 30 minutes.

**Supplemental Table 10.** Physical activity levels pre-, 6-months post-diagnosis and change in physical activity, overall, by race, and age (N=2994).

|  | All ages | | <50 | | | | ≥50 | | | |
| --- | --- | --- | --- | --- | --- | --- | --- | --- | --- | --- |
|  | CBCS 3 population  N=2994 | | Non-Black  N=750 | | Black  N=739 | | Non-Black  N=752 | | Black  N=753 | |
| Pre-diagnosis |  |  |  | |  | |  | |  | |
|  | **N** | **%** | **N** | **%** | **N** | **%** | **N** | **%** | **N** | **%** |
| Sufficient MVPA(A) | 1845 | 61.7 | 487 | 64.9 | 443 | 60.2 | 485 | 64.7 | 430 | 57.1 |
| Insufficient MVPA(B) | 663 | 22.2 | 156 | 20.8 | 177 | 24.0 | 155 | 20.7 | 175 | 23.2 |
| No MVPA (C) | 480 | 16.1 | 107 | 14.3 | 116 | 15.8 | 109 | 14.5 | 148 | 19.6 |
| 6-month post- diagnosis |  |  |  | |  | |  | |  | |
| Sufficient MVPA (A) | 956 | 32.0 | 303 | 40.4 | 166 | 22.5 | 292 | 38.9 | 195 | 25.9 |
| Insufficient MVPA(B) | 701 | 23.4 | 187 | 24.9 | 171 | 23.2 | 171 | 22.8 | 172 | 22.8 |
| No MVPA (C) | 1334 | 44.6 | 260 | 34.7 | 400 | 54.3 | 288 | 38.3 | 386 | 51.3 |
| Change between pre- and post-diagnosis |  |  |  | |  | |  | |  | |
| Decreased |  |  |  |  |  |  |  |  |  |  |
| A🡪C: | 683 | 22.9 | 136 | 18.1 | 217 | 29.5 | 143 | 19.1 | 187 | 24.8 |
| A🡪B: | 392 | 13.1 | 111 | 14.8 | 97 | 13.2 | 94 | 12.6 | 90 | 11.9 |
| B🡪C: | 326 | 10.9 | 66 | 8.8 | 96 | 13.1 | 69 | 9.2 | 95 | 12.6 |
| Increased |  |  |  |  |  |  |  |  |  |  |
| C🡪B: | 87 | 2.9 | 25 | 3.3 | 17 | 2.3 | 20 | 2.7 | 25 | 3.3 |
| C🡪A: | 70 | 2.3 | 24 | 3.2 | 14 | 1.9 | 13 | 1.7 | 19 | 2.5 |
| B🡪A: | 116 | 3.9 | 24 | 3.2 | 24 | 3.3 | 30 | 4.0 | 23 | 3.0 |
| Unchanged PA levels |  |  |  |  |  |  |  |  |  |  |
| Remained no MVPA: | 323 | 10.9 | 58 | 7.7 | 85 | 11.6 | 76 | 10.2 | 104 | 13.8 |
| Remained insufficient MVPA: | 221 | 7.4 | 51 | 6.8 | 57 | 7.8 | 56 | 7.5 | 57 | 7.6 |
| Remained Sufficient MVPA: | 768 | 25.7 | 240 | 32.0 | 128 | 17.4 | 247 | 33.0 | 153 | 20.3 |
| Missing | 8 |  |  |  | 4 |  |  | 4 |  |  |

**Supplemental Table 11.** Associations between demographic correlates and 6-month post-diagnosis and change in MVPA.^§^

|  | 6-month Post-Diagnosis MVPA | | Change in MVPA | |
| --- | --- | --- | --- | --- |
|  | Any MVPA vs No MVPA | p-value | Increased or no change in MVPA vs decreased MVPA | p-value |
| Race |  |  |  |  |
| Black | 1. |  | 1. |  |
| Non-Black | 1.72 (1.47, 2.01) | **<0.0001** | 1.58 (1.30, 1.92) | **<0.0001** |
|  |  |  |  |  |
| Age at diagnosis |  |  |  |  |
| <50 | 1. |  | 1. |  |
| ≥50 | 0.84 (0.72, 0.98) | **0.04** | 1.09 (0.89, 1.32) | 0.32 |
|  |  |  |  |  |
| Income |  |  |  |  |
| ≤15K | 1. |  | 1. |  |
| >15K-≤30K | 1.09 (0.86, 1.39) | 0.55 | 0.90 (0.66, 1.23) | 0.95 |
| >30K | 1.75 (1.44, 2.13) | **<0.0001** | 1.40 (1.09, 1.80) | **0.003** |
|  |  |  |  |  |
| Education |  |  |  |  |
| <= HS Graduate | 1. |  | 1. |  |
| >= Some college | 1.69 (1.44, 1.98) | **<0.0001** | 1.45 (1.19, 1.77) | **0.0008** |

§Analysis using multivariable-adjusted polytomous model to calculate odds ratios and 95% CI (N=2994).

Abbreviations: CI= confidence interval; MVPA= moderate to vigorous physical activity; HS= high school

a. Multivariable adjusted model includes adjustment for minutes per week of physical activity pre-diagnosis, age at diagnosis, endocrine therapy, lymphedema, stage, and chemotherapy.

b. To evaluate changes between pre-diagnosis and 6-month post-diagnosis MVPA, the categorical variable is defined as follows: **i**ncreased activity by > 30 minutes; decreased activity by > 30 minutes; no change, within 30 minutes.

**Supplemental Table 12.** Social level correlates associated with 6-month post-diagnosis and change in MVPA.^§^

|  | 6-month Post-Diagnosis MVPA | | Change in MVPA | |
| --- | --- | --- | --- | --- |
|  | Any MVPA vs No MVPA | p-value | Increased or no change in MVPA vs decreased MVPA | p-value |
| Access to healthcare |  |  |  |  |
| More barriers | 1. |  | 1. |  |
| Fewer barriers | 1.21 (1.01, 1.45) | **0.05** | 1.23 (0.98, 1.55) | **0.052** |
|  |  |  |  |  |
| Community level SES |  |  |  |  |
| Deprivation |  |  |  |  |
| High | 1. |  | 1. |  |
| Low | 1.45 (1.24, 1.70) | **<0.0001** | 1.49 (1.21, 1.84) | **0.0001** |
|  |  |  |  |  |
| Assets |  |  |  |  |
| Low | 1. |  | 1. |  |
| High | 1.54 (1.31, 1.79) | **<0.0001** | 1.46 (1.21, 1.77) | **0.0002** |

§Analysis using multivariable-adjusted polytomous model to calculate odds ratios and 95% CI (N=2994).

Abbreviations: CI= confidence interval; MVPA= moderate to vigorous physical activity; SES = socioeconomic status.

a. Multivariable adjusted model includes adjustment for minutes per week of physical activity pre-diagnosis, age at diagnosis, endocrine therapy, lymphedema, stage, and chemotherapy.

b. To evaluate changes between pre-diagnosis and 6-month post-diagnosis MVPA, the categorical variable is defined as follows: increased activity by > 30 minutes; decreased activity by > 30 minutes; no change, within 30 minutes.

c. Access to healthcare (fewer vs more barriers) was defined by insurance, urban/rural residence, job loss, and transportation issues.

d. Deprivation includes no vehicle, public assistance, unemployment, crowded housing, renting, single householder, female householder, and below poverty line whereas assets include Male/female professionals.

**Supplemental Table 13.** Clinical correlates associated with 6-month post-diagnosis and change in MVPA.^§^

|  | 6-month Post-Diagnosis MVPA | | Change in MVPA | |
| --- | --- | --- | --- | --- |
|  | Any MVPA vs No MVPA | p-value | Increased or no change in MVPA vs decreased MVPA | p-value |
| BMI |  |  |  |  |
| ≥30 | 1. |  | 1. |  |
| 25 - <30 | 1.28 (1.06, 1.53) | **0.01** | 1.25 (0.99, 1.56) | **0.03** |
| <25 | 1.67 (1.38, 2.04) | **<0.0001** | 1.69 (1.34, 2.15) | **0.0001** |
|  |  |  |  |  |
| Endocrine therapy |  |  |  |  |
| No | 1. |  | 1. |  |
| Yes | 1.49 (1.27, 1.76) | **<0.0001** | 1.21 (1.00, 1.49) | **0.05** |
|  |  |  |  |  |
| Chemotherapy |  |  |  |  |
| Yes | 1. |  | 1. |  |
| No | 1.46 (1.20, 1.77) | **<0.0001** | 1.78 (1.41, 2.26) | **<0.0001** |
|  |  |  |  |  |
| Lymphedema |  |  |  |  |
| Yes | 1. |  | 1. |  |
| No | 1.27 (0.92, 1.75) | 0.13 | 1.26 (0.83, 1.92) | 0.20 |
|  |  |  |  |  |
| Stage |  |  |  |  |
| 3 or 4 | 1. |  | 1. |  |
| 1 | 1.37 (1.08, 1.75) | **0.02** | 0.97 (0.72, 1.31) | 0.98 |
| 2 | 1.05 (0.85, 1.31) | 0.81 | 0.91 (0.69, 1.19) | 0.62 |

§Analysis using multivariable-adjusted polytomous model to calculate odds ratios and 95% CI (N=2940).

Abbreviations: CI= confidence interval; MVPA= moderate to vigorous physical activity; BMI=body mass index;

a. Multivariable adjusted model includes adjustment for minutes per week of physical activity pre-diagnosis, age at diagnosis, endocrine therapy, lymphedema, stage, and chemotherapy.

b. To evaluate changes between pre-diagnosis and 6-month post-diagnosis MVPA, the categorical variable is defined as follows: increased activity by > 30 minutes; decreased activity by > 30 minutes; no change, within 30 minutes.

c. Participants who did not receive surgery were excluded (n=54).

**Supplemental Table 14.** Associations between demographic correlates and 18-month post-diagnosis.^§^

|  | 18-month Post-Diagnosis MVPA | |
| --- | --- | --- |
|  | Any MVPA vs No MVPA | p-value |
| Race |  |  |
| Black | 1. |  |
| Non-Black | 1.12 (0.89, 1.39) | 0.37 |
|  |  |  |
| Age at diagnosis |  |  |
| <50 | 1. |  |
| ≥50 | 0.78 (0.63, 0.98) | **0.03** |
|  |  |  |
| Income |  |  |
| ≤15K | 1. |  |
| >15K-≤30K | 0.99 (0.72, 1.37) | 0.86 |
| >30K | 1.33 (1.02, 1.74) | **0.03** |
|  |  |  |
| Education |  |  |
| <= HS Graduate | 1. |  |
| >= Some college | 1.61 (1.27, 2.03) | **0.0001** |

§Analysis using multivariable-adjusted polytomous model to calculate odds ratios and 95% CI (N=2994).

Abbreviations: CI= confidence interval; MVPA= moderate to vigorous physical activity; HS= high school

a. Multivariable adjusted model includes adjustment for minutes per week of physical activity pre-diagnosis, age at diagnosis, endocrine therapy, lymphedema, stage, and chemotherapy.

**Supplemental Table 15.** Social level correlates associated with 18-month post-diagnosis.^§^

|  | 18-month Post-Diagnosis MVPA | |
| --- | --- | --- |
|  | Any MVPA vs No MVPA | p-value |
| Access to healthcare |  |  |
| More barriers | 1. |  |
| Fewer barriers | 1.19 (0.93, 1.53) | 0.15 |
|  |  |  |
| Community level SES |  |  |
| Deprivation |  |  |
| High | 1. |  |
| Low | 1.34 (1.08, 1.67) | **0.008** |
|  |  |  |
| Assets |  |  |
| Low | 1. |  |
| High | 1.55 (1.23, 1.94) | **0.0002** |

§Analysis using multivariable-adjusted polytomous model to calculate odds ratios and 95% CI (N=2994).

Abbreviations: CI= confidence interval; MVPA= moderate to vigorous physical activity; SES = socioeconomic status.

a. Multivariable adjusted model includes adjustment for minutes per week of physical activity pre-diagnosis, age at diagnosis, endocrine therapy, lymphedema, stage, and chemotherapy.

b. Access to healthcare (fewer vs more barriers) was defined by insurance, urban/rural residence, job loss, and transportation issues.

c. Deprivation includes no vehicle, public assistance, unemployment, crowded housing, renting, single householder, female householder, and below poverty line whereas assets include Male/female professionals.

**Supplemental Table 16.** Clinical correlates associated with 18-month post-diagnosis.^§^

|  | 18-month Post-Diagnosis MVPA | |
| --- | --- | --- |
|  | Any MVPA vs No MVPA | p-value |
| BMI |  |  |
| ≥30 | 1. |  |
| 25 - <30 | 1.09 (0.85, 1.41) | 0.49 |
| <25 | 1.30 (0.98, 1.73) | 0.07 |
|  |  |  |
| Endocrine therapy |  |  |
| No | 1. |  |
| Yes | 1.15 (0.91, 1.46) | 0.25 |
|  |  |  |
| Chemotherapy |  |  |
| Yes | 1. |  |
| No | 0.69 (0.53, 0.91) | **0.007** |
|  |  |  |
| Lymphedema |  |  |
| Yes | 1. |  |
| No | 1.05 (0.67, 1.66) | 0.81 |
|  |  |  |
| Stage |  |  |
| 3 or 4 | 1. |  |
| 1 | 1.72 (1.22, 2.43) | **0.002** |
| 2 | 1.34 (0.99, 1.83) | **0.053** |

§Analysis using multivariable-adjusted polytomous model to calculate odds ratios and 95% CI (N=2940).

Abbreviations: CI= confidence interval; MVPA= moderate to vigorous physical activity; BMI=body mass index;

a. Multivariable adjusted model includes adjustment for minutes per week of physical activity pre-diagnosis, age at diagnosis, endocrine therapy, lymphedema, stage, and chemotherapy.

b. Participants who did not receive surgery were excluded (n=54).
